# SERS Taper-Fiber Nanoprobe Modified by Gold Nanoparticles Wrapped with Ultrathin Alumina Film by Atomic Layer Deposition

**DOI:** 10.3390/s17030467

**Published:** 2017-02-25

**Authors:** Wenjie Xu, Zhenyi Chen, Na Chen, Heng Zhang, Shupeng Liu, Xinmao Hu, Jianxiang Wen, Tingyun Wang

**Affiliations:** Key Laboratory of Specialty Fiber Optics and Optical Access Networks, School of Communication and Information Engineering, Shanghai University, 333 Nanchen Road, Shanghai 200444, China; wenjiexu@163.com (W.X.); zychen@shu.edu.cn (Z.C.); zhcn_2010@163.com (H.Z.); liusp@shu.edu.cn (S.L.); cmol_hu@shu.edu.cn (X.H.); wenjx@shu.edu.cn (J.W.); tywang@shu.edu.cn (T.W.)

**Keywords:** taper-fiber nanoprobe, gold nanoparticles, atomic layer deposition, surface enhancement Raman spectroscopy

## Abstract

A taper-fiber SERS nanoprobe modified by gold nanoparticles (Au-NPs) with ultrathin alumina layers was fabricated and its ability to perform remote Raman detection was demonstrated. The taper-fiber nanoprobe (TFNP) with a nanoscale tip size under 80 nm was made by heated pulling combined with the chemical etching method. The Au-NPs were deposited on the TFNP surface with the electrostatic self-assembly technology, and then the TFNP was wrapped with ultrathin alumina layers by the atomic layer deposition (ALD) technique. The results told us that with the increasing thickness of the alumina film, the Raman signals decreased. With approximately 1 nm alumina film, the remote detection limit for R6G aqueous solution reached 10^−6^ mol/L.

## 1. Introduction

Serving as an attractive analytical spectral technique, Raman spectroscopy provides a “fingerprint” of information of molecular structures [1,2]. Unfortunately, due to its relatively low Raman scattering cross-section (~10^−30^ cm^2^ per molecule) [3] and being hidden in fluorescence spectroscopy [4], the original Raman scattering is extremely weak and its potential applications were limited, until surface enhancement Raman spectroscopy (SERS), which paved the way for the extension of Raman spectroscopy to the realm of trace detection [5], was discovered in 1974 [6]. SERS can strongly increase the Raman signals of a molecule attached to nanoscale noble metallic structures, such as gold, silver and copper, etc. [7,8,9]. Among the surfaces of these noble metals, the localized surface plasma and the Raman signals will appear as resonance, and then the Raman signals will be extremely enhanced. In addition, the morphology of these noble metal nanostructures will significantly influence the SERS effect, leading to various morphological substrates investigated and novel nanostructures proposed [10,11,12]. A single-molecule detection was demonstrated by the SERS technique in different environments [13], even in single living cells [14].

Optical fibers, as sensors, have been used for SERS to detect molecules in remote locations and biomedical applications [15]. This group of optical fiber sensors, whose remote capabilities are often called “optrode” [5], can be classified into two categories by the Raman signals collected either in backward scattering through the same fiber or in forward scattering by the other separated fiber. There are types of optical fiber structures serving as SERS detectors: the liquid-core photonic crystal fiber probe (LCPCF) prepared to detect the 10^−10^ mol/L R6G solution mixed with nanoparticles (NPs) [16], the nanopillar array on a fiber facet for in situ remote SERS sensing of toluene vapor [17], fiber tips with angled polishing [18], flat fiber tips ablated by a femtosecond laser [19], and the taper-fiber probe with silver nanoplates fabricated to detect the 10^−7^ mol/L 4-ATP [20]. The taper-fiber tips with an optimal cone angle can achieve superior performance for an optical fiber SERS detection, due to increasing the SERS active surface [21], transmitting the evanescent field to the surroundings with an increased magnitude [22], and good collection efficiency [23]. Nevertheless, the SERS substrates may be easily damaged by exposure to air or samples, and will not remain stable for a long time.

The proposal of shell-isolated nanoparticle-enhanced Raman spectroscopy [24] was generally accepted to be a milestone in Raman spectroscopy. Noble metal NPs coated with metal oxide layers were reported to be able to suppress metal surface reactions with air, and preserve their morphology at high temperatures for an extended period of time without losing their enhancement capabilities [25,26,27,28]. These coated structures can be applied to complex environment detection and survive with long-term stability [29,30]. It was indicated, in some literatures, that an Ag island film coated with protected alumina was able to establish a reusable and stable SERS substrate [31], and Ag nanorods wrapped with ultrathin alumina layers were able to stabilize their SERS sensitivity in air for more than 50 days [30]. Besides, nanostructures with ultrathin (~1.5 nm) layers will not decrease the SERS performance much. Hence, it is highly desirable to wrap our SERS nanoprobe with ultrathin alumina layers.

In this literature, based on our previous work [32], we prepared a taper-fiber nanoprobe (TFNP) modified by Au-NPs, and wrapped it with ultrathin alumina film. The nanoscale taper-fiber probe tip was made through the processes of heated pulling combined with chemical etching. Electrostatic self-assembly technology was adopted to deposit Au-NPs on the tip surface of the TFNP, and then it was wrapped with ultrathin alumina film via the ALD technique “layer by layer”. Direct measurement to R6G adsorbed on Au-NPs with different alumina layers on this probe surface was made. Raman spectra of R6G aqueous solutions with different concentrations were detected in the remote detection mode by this fiber SERS nanoprobe.

## 2. Experiment Procedures and Methods

### 2.1. Fabrication of the Nanoscale Taper-Fiber

**Figure 1:**
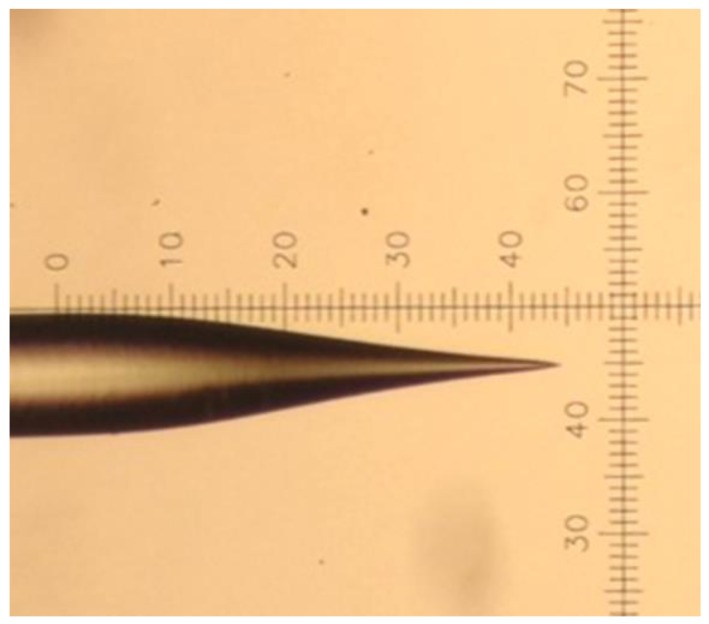
Optical microscope photographs of taper-fiber nanoprobe with 10× objective.

A multimode fiber with a core/cladding diameter of 50/125 µm was used in our experiment. The nanoscale taper-fiber was obtained through the heated pulling method combined with chemical etching [33,34,35]. The specific process is the same as our previous works [32]. After etching, the fiber was rinsed with deionized water. The final result of this taper-fiber tip is shown in Figure 1.

The taper-fiber has a very sharp tip and its angle is about 30°. It is illustrated that the cone length of this tip is measured at around 330 μm. Generally, taper-fiber probe with larger taper angle and shorter taper cone length is more efficient to collect scattering light. However, in the applications of puncture measurements, smaller taper angle is required, so with the integrative considerations, the taper angle should be a trade-off in some cases.

### 2.2. Preparation of Gold NPs

Analogy to our previous works [32], Gold colloids were still prepared with the classic Frenz [36] method, for its being rapid and uniform. The preparation procedures were repeated as follows: 100 mL 0.01% (w) chloroauric acid (HAuCl_4_) solution prepared in a beaker, and then heated to boiling; 0.5 mL 1% (w) trisodium citrate (Na_3_C_6_H_5_O_7_·2H_2_O) solution, which makes Au-NPs 55 nm in average diameter, was added to the boiling HAuCl_4_ solution and kept the mixture solution boiling state for 5 min. That Au-NPs with diameters between 50 nm and 60 nm have the optimal enhancement effect for the R6G’s Raman spectra detection has been demonstrated [37].

### 2.3. Modified Tapered Fiber Nanoprobe with Gold NPs

Although it is time-consuming, electrostatic self-assembly technology, due to it making Au-NPs uniform on the taper-fiber tips, is still adopted to form the SERS-active substrate, as our previous works [32].

Firstly, the taper-fiber tip should be totally cleaned by immersing into a piranha solution (3:1 mixture of 96% concentrated sulfuric acid and 30% hydrogen peroxide) for 30 min, and then rinsing twice in deionized water and ethanol respectively. Secondly, the cleaned fiber was soaked in the mixture solution with 5% v/v deionized water, 5% v/v (3-Aminopropyl) trimethoxysilane (APTMS 97%) and 90% ethanol for 30 min. Again, it was rinsed twice in deionized water and ethanol respectively to remove the residual APTMS. Thirdly, it was kept in the incubator at 90 °C for 30 min. Finally, this fiber was cleaned in the ethanol and then immersed into the gold colloids for 48 h.

**Figure 2:**
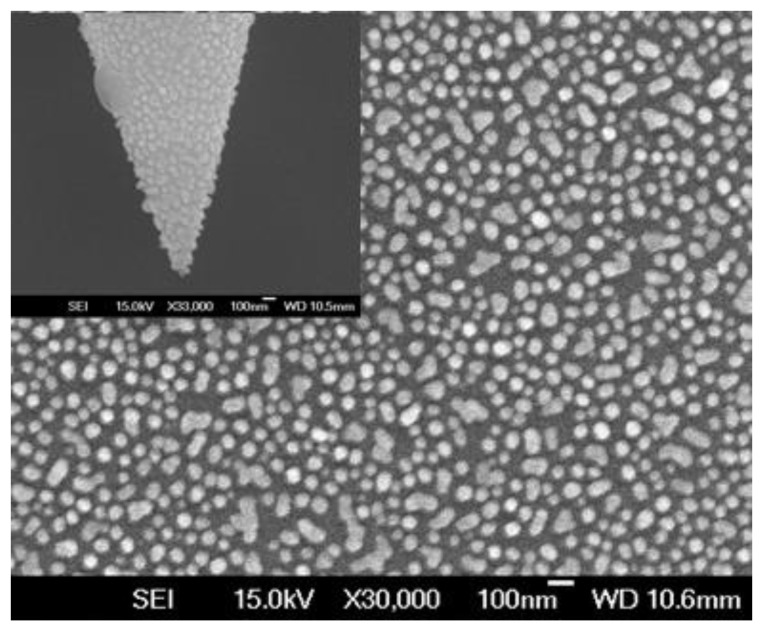
SEM photographs of the distribution of Au-NPs on the TFNP surface and nanoprobe tip inset.

**Figure 3:**
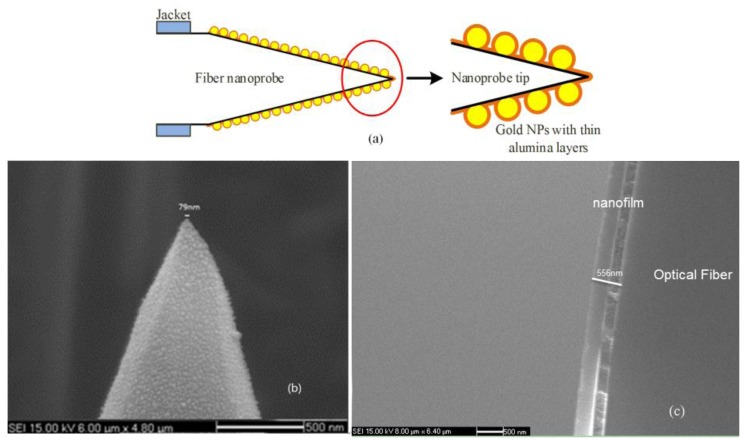
(**a**) Profile illustration of Au-NPs modified TFNP with ultrathin alumina layers; (**b**) SEM photograph of Au-NPs modified TFNP with ultrathin alumina layers (with 10 cycles); (**c**) SEM photograph of fiber on which deposited alumina film by ALD technique with 5000 cycles (first 2000 cycles and then 3000 cycles).

The morphology photographs of our TFNP modified by Au-NPs were obtained by scanning electron microscopy (SEM) (Figure 2). As expected, the Au-NPs were uniformly distributed on the surface of TFNP, and the nanoprobe has a tip dimension under 80 nm (Figure 3b) which have great potential in puncture measurements.

### 2.4. Wrap the Modified Nanoprobe with Alumina Layers

The taper-fiber nanoprobes prepared above, in puncture measurement, were used to detect the intracellular Raman signals but the corresponding biologic Raman signals were very weak and even sometimes no biologic Raman signal was detected. Therefore, we assumed that due to the friction and collision between nanoparticles and cell membrane, the dropping and surface morphology changing of the AuNPs might cause the signals weak and even disappearing. In order to gain better potential for puncture biosensing such as intracellular, we wrap the modified nanoprobe with alumina layers, which can sacrifice itself to protecting the Raman enhancement substrates from being damaged or dropping in the process of piercing the cell or other analytes. The alumina nano-layers were deposited on the gold modified TFNP with the ALD equipment (TFS 200, Beneq, Espoo, Finland). There is a disc shape substrate with diameter 22 cm and the height of 3 cm in its reaction chamber. Using high purity nitrogen as the carrier and purge gas, two precursors, trimethyl aluminum (TMA) and water vapors, were valve controlled and carried into the heated chamber (about 210 °C) by the way of pulse alternatively. Quintessentially, one reaction cycle consisted of four steps: (1) TMA reactant adsorbed on the surface of sample; (2) nitrogen purging; (3) water vapor exposure in the chamber and reaction with TMA; (4) nitrogen purging. One reaction cycle completed, the monolayer Al_2_O_3_ was deposited on the sample-surface. Through repeating the deposition cycle, Al_2_O_3_ film grows thicker layer by layer and its thickness can be precisely controlled by altering the number of cycles.

As to our ALD equipment, one technique corresponds to one thickness of the film in a deposition cycle. In order to confirm the thickness of the film per cycle in our alumina deposition, we have deposited 300 ALD cycles on the clean silicon wafer, and the total thickness of the arbitrary six points of the coated film was measured by ellipsometer, and the results are shown in Table 1. It was demonstrated that the thickness was uniform, the refractive index of alumina film was 1.658, and the deposition rate is approximate 0.12 nm/cycle. We also have deposited 5000 ALD cycles on the clean surface of fiber by two steps (first 2000 cycles and then 3000 cycles), and its SEM photograph is shown in Figure 3c. The SEM photograph told us that an average deposition rate is approximate 0.1112 nm/cycle. Considering above data, we finally regarded 0.12 nm/cycle as the deposition rate. By this approach, our gold modified nanoprobe was coated with eight ALD cycles (~1 nm). The diagram representing profile of this after-wrapped nanoprobe illustrates in Figure 3a.

### 2.5. SERS Measurements

**Figure 4:**
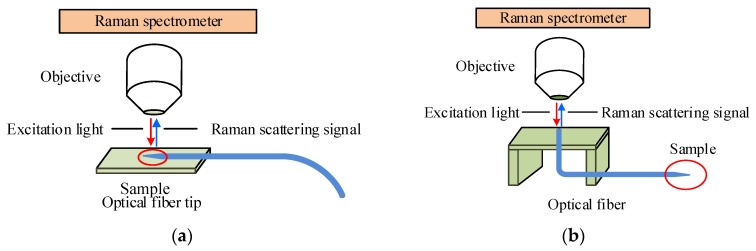
Experimental setup for (**a**) direct measurement and (**b**) detection in optrode configuration.

Rhodamine 6G (R6G) aqueous solution was used as the standard sample, to ascertain the SERS performances of the TFNP with ultrathin alumina layers. The Raman spectra of R6G were detected by the above prepared fiber SERS nanoprobe with a commercial confocal micro-Raman spectrometer (Renishaw inVia plus) by direct measurement and in optrode configuration. The experimental setups illustrate in Figure 4.

By direct measurement, the tip of TFNP was put on an object stage (Figure 4a), adjusting the objective to optimum spot for measurement. Using 633 nm He-Ne laser as exciting light, whose power is around 2 mW ahead objective, the SERS enhancement effect of TFNP tip surface including Au-NPs wrapped with different alumina layers was investigated. The microscope objective used 50× for high efficient collecting SERS signal from the tip surface of TFNP. R6G aqueous solution with a concentration of 10^−6^ mol/L was used as the detection sample.

In optrode configuration, the fiber SERS nanoprobe was usually cut into segments of 15–20 cm with a flat end surface for laser injection. Through the 10× microscope objective for matching the NA of fiber, 633 nm He-Ne laser with the power of approximate 2 mW was injected into the fiber from its flat end surface. The backward SERS signal of the R6G molecules on the nanoprobe surface was collected and transmitted by the same nanoprobe, coupled to the same “injection” objective, finally entered the Raman spectrometer (Figure 4b). The exposure time of Raman spectrometer was set to 10 s for each measurement. All the experimental conditions were the same in the whole measurement, to guarantee the results believable.

## 3. Results and Discussions

**Figure 5:**
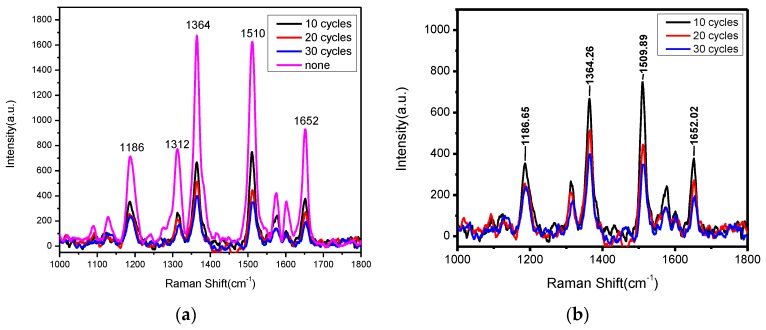
(**a**) Comparison of Raman spectra of R6G (10^−6^ mol/L) adsorbed on gold NPs coated with and without alumina layers; (**b**) Raman spectra of R6G (10^−6^ mol/L) adsorbed on gold NPs coated with different alumina layers.

The direct measurement results are shown in Figure 5, with the baseline subtracted and the Savitzky-Golay filter applied for clarity and to increase the signal-to-noise ratio without greatly distorting the signal [38], respectively. For each spectrum the baseline is the broken line composed of a series of segments, which is obtained by connecting the two lowest points of each Raman peak [32].

Obviously, all coated Au-NPs exhibited good SERS sensitivity, and the Raman peaks of the R6G molecule were marked in the two figures, with the peaks’ position difference from the data process errors. Compared with the pink line, which represents Au-NPs without an alumina layer, in Figure 5a, the Raman peak intensity of the Au-NPs wrapped with alumina layers (~1.2 nm) was lower by around half. As a main contributor to the enhancements of electromagnetics, Au-NPs were imprisoned by alumina shells and their performance of enhancement was limited. In addition, the Raman spectra of Au-NPs with different thicknesses of alumina layers are more clearly compared in Figure 5b. As expected, the Raman signal intensity decreased with the increase of the alumina thickness.

**Figure 6:**
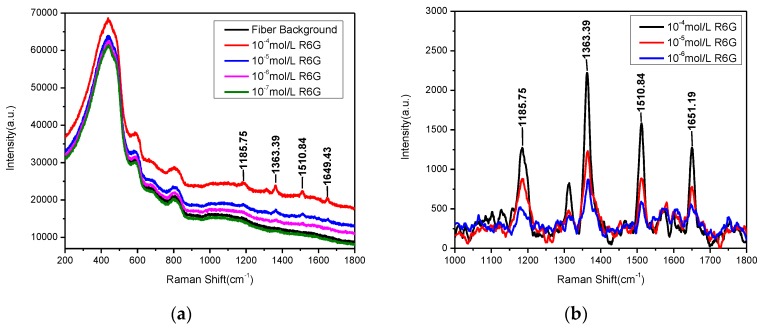
(**a**) SERS spectra of R6G with different concentrations in remote mode; (**b**) Baseline subtracted from (**a**).

In order to ascertain the remote sensing potential of the TFNP with ultrathin alumina protective film, Raman spectra of the fiber itself and R6G aqueous solutions with four different concentrations (10^−4^ mol/L, 10^−5^ mol/L, 10^−6^ mol/L, 10^−7^ mol/L) were measured in optrode configuration, shown in Figure 6a. Raman spectra, except those of the fiber itself and the lowest concentration of the R6G (10^−7^ mol/L) aqueous solution due to them being too low to detect, from 1000 cm^−1^ to 1800 cm^−1^ with the baselines subtracted and the Savitzky-Golay filter applied, are shown in Figure 6b. It is obviously demonstrated that our nanoprobes possess preferable remote sensing potential and Raman intensity in each peak position which became weaker and weaker with the decrease of the R6G concentration. Furthermore, four Raman characteristic peaks (1186 cm^−1^, 1363 cm^−1^, 1510 cm^−1^, 1651 cm^−1^) can still be recognized at the minimum concentration of 10^−6^ mol/L. Due to being wrapped with a layer of ultrathin alumina film, the detection limit for R6G aqueous solution only reached 10^−6^ mol/L in the optrode configuration, two orders of magnitude lower than in our previous work [32] (with a limit of 10^−8^ mol/L). It is obviously shown, from these remote sensing results, that this kind of fiber SERS nanoprobe with ultrathin alumina layers (~1 nm) still possesses a good enhancement effect and the NPs would be protected by the alumina film for reusability and puncture measurements. Besides, the fiber background in our experiment is still strong (Figure 6a), and therefore decreasing the peak of the background will be one of the future research aspects.

## 4. Conclusions

We proposed a nanoscale taper-fiber SERS probe structure modified by Au-NPs wrapped with alumina ultrathin film and presented its fabrication method. Its SERS performance in the optrode configuration was demonstrated in experiments. The taper-fiber probe with a tip size under 80 nm was realized through heated pulling and chemical etching methods, with Au-NPs deposited on the nanoprobe surface by electrostatic self-assembly technology; the nanoprobe was wrapped with ultrathin alumina layers using the ALD technique. It is indicated that in the same concentration of R6G aqueous solution, the alumina layers were thicker and the Raman spectra enhancement effect was weaker due to the Au-NPs imprisoned by the thick alumina layers. Also, with approximately 1 nm alumina layers, the detection limit of the R6G aqueous solution reached 10^−6^ mol/L in the optrode configuration. The nanoscale, remote sensing SERS-active nanoprobe, wrapped with ultrathin alumina layers for protecting the AuNPs, may have great potential for puncture biosensing such as intracellular applications. An a great deal of effort should be devoted to suppressing the background Raman scattering of the fiber itself to get the higher signal-to-noise ratio and long-distance sensing capabilities.

## Figures and Tables

**Table 1:** Thickness of the film on the silicon wafer in 6 different points.

Position	Thickness (nm)	N (633 nm)
#1	36.618	1.658
#2	37.083	1.658
#3	37.822	1.658
#4	36.672	1.658
#5	37.021	1.658
#6	37.706	1.658

## References

[1] Tamor M.A., Vassell W.C. (1994). Raman “fingerprinting” of amorphous carbon films. J. Appl. Phys..

[2] Cao Y.W.C., Jin R., Mirkin C.A. (2002). Nanoparticles with Raman spectroscopic fingerprints for DNA and RNA detection. Science.

[3] Nie S., Emory S.R. (1997). Probing single molecules and single nanoparticles by surface-enhanced Raman scattering. Science.

[4] Gessner R., Rösch P., Petry R., Schmitt M., Strehle M.A., Kiefer W., Popp J. (2004). The application of a SERS fiber probe for the investigation of sensitive biological samples. Analyst.

[5] Andrade G.F.S., Fan M.K., Brolo A.G. (2010). Multilayer silver nanoparticles-modified optical fiber tip for high performance SERS remote sensing. Biosens. Bioelectron..

[6] Fleischmann M., Hendra P.J., McQuillan A. (1974). Raman spectra of pyridine adsorbed at a silver electrode. Chem. Phys. Lett..

[7] Nguyen C.T., Nguyen J.T., Rutledge S., Zhang J., Wang C., Walker G.C. (2010). Detection of chronic lymphocytic leukemia cell surface markers using surface enhanced Raman scattering gold nanoparticles. Cancer Lett..

[8] Mulvihill M.J., Ling X.Y., Henzie J., Yang P. (2009). Anisotropic etching of silver nanoparticles for plasmonic structures capable of single-particle SERS. J. Am. Chem. Soc..

[9] Shao Q., Que R., Shao M., Cheng L., Lee S.T. (2012). Copper Nanoparticles Grafted on a Silicon Wafer and Their Excellent Surface-Enhanced Raman Scattering. Adv. Funct. Mater..

[10] Lin X.M., Cui Y., Xu Y.H., Ren B., Tian Z.Q. (2009). Surface-enhanced Raman spectroscopy: substrate-related issues. Anal. Bioanal. Chem..

[11] Betz J.F., Wei W.Y., Cheng Y., White I.M., Rubloff G.W. (2014). Simple SERS substrates: Powerful, portable, and full of potential. Phys. Chem. Chem. Phys..

[12] Schlücker S. (2014). Surface-Enhanced Raman spectroscopy: Concepts and chemical applications. Angew. Chem. Int. Ed..

[13] Pieczonka N.P.W., Aroca R.F. (2008). Single molecule analysis by surfaced-enhanced Raman scattering. Chem. Soc. Rev..

[14] Scaffidi J.P., Gregas M.K., Seewaldt V., Vo-Dinh T. (2009). SERS-based plasmonic nanobiosensing in single living cells. Anal. Bioanal. Chem..

[15] Stoddart P.R., White D.J. (2009). Optical fibre SERS sensors. Anal. Bioanal. Chem..

[16] Zhang Y., Shi C., Gu C., Seballos L., Zhang J.Z. (2007). Liquid core photonic crystal fiber sensor based on surface enhanced Raman scattering. Appl. Phys. Lett..

[17] Yang X., Ileri N., Larson C.C., Carlson T.C., Britten J.A., Chang A.S., Bond T.C. (2012). Nanopillar array on a fiber facet for highly sensitive surface-enhanced Raman scattering. Opt. Express.

[18] Viets C., Hill W. (2000). Single-fibre surface-enhanced Raman sensors with angled tips. J. Raman Spectrosc..

[19] Lan X., Han Y., Wei T., Zhang Y., Jiang L., Tsai H.L., Xiao H. (2009). Surface-enhanced Raman-scattering fiber probe fabricated by femtosecond laser. Opt. Lett..

[20] Cao J., Zhao D., Lei X., Liu Y., Mao Q. (2014). One-pot hydrothermal synthesis of silver nanoplates on optical fiber tip for surface-enhanced Raman scattering. Appl. Phys. Lett..

[21] Lucotti A., Zerbi G. (2007). Fiber-optic SERS sensor with optimized geometry. Sens. Actuators B Chem..

[22] Sharma A.K., Jha R., Gupta B.D. (2007). Fiber-optic sensors based on surface plasmon resonance: A comprehensive review. IEEE Sens. J..

[23] Jayawardhana S., Mazzolini A.P., Stoddart P.R. (2012). Collection efficiency of scattered light in single-ended optical fiber sensors. Opt. Lett..

[24] Li J.F., Huang Y.F., Ding Y., Yang Z.L., Li S.B., Zhou X.S., Fan F.R., Zhang W., Zhong Z.Y., Wu D.Y. (2010). Shell-isolated nanoparticle-enhanced Raman spectroscopy. Nature.

[25] Whitney A.V., Elam J.W., Stair P.C., Van Duyne R.P. (2007). Toward a thermally robust operando surface-enhanced Raman spectroscopy substrate. J. Phys. Chem. C.

[26] Formo E.V., Mahurin S.M., Dai S. (2010). Robust SERS substrates generated by coupling a bottom-up approach and atomic layer deposition. ACS Appl. Mater. Interfaces.

[27] John J.F., Mahurin S., Dai S., Sepaniak M.J. (2010). Use of atomic layer deposition to improve the stability of silver substrates for in situ, high-temperature SERS measurements. J. Raman Spectrosc..

[28] Bachenheimer L., Elliott P., Stagon S., Huang H. (2014). Enhanced thermal stability of Ag nanorods through capping. Appl. Phys. Lett..

[29] Zhang W., Dong J.C., Li C.Y., Chen S., Zhan C., Panneerselvam R., Yang Z.L., Li J.F., Zhou Y.L. (2015). Large scale synthesis of pinhole-free shell-isolated nanoparticles (SHINs) using improved atomic layer deposition (ALD) method for practical applications. J. Raman Spectrosc..

[30] Ma L., Huang Y., Hou M., Xie Z., Zhang Z.J. (2015). Silver nanorods wrapped with ultrathin Al2O3 layers exhibiting excellent SERS sensitivity and outstanding SERS stability. Sci. Rep..

[31] Mahurin S.M., John J., Sepaniak M.J., Dai S. (2011). A reusable surface-enhanced Raman scattering (SERS) substrate prepared by atomic layer deposition of alumina on a multi-layer gold and silver film. Appl. Spectrosc..

[32] Chen Z.Y., Dai Z.M., Chen N., Liu S.P., Pang F.F., Lu B., Wang T.Y. (2014). Gold nanoparticles-modified tapered fiber nanoprobe for remote SERS detection. IEEE Photonics Technol. Lett..

[33] Lazarev A., Fang N., Luo Q. (2003). Formation of fine near-field scanning optical microscopy tips. Part II. By laser heated pulling and bending. Rev. Sci. Instrum..

[34] Lazarev A., Fang N., Luo Q., Zhang X. (2003). Formation of fine near-field scanning optical microscopy tips. Part I. By static and dynamic chemical etching. Rev. Sci. Instrum..

[35] Gu N., Li C., Sun L., Liu Z.H., Sun Y.K., Xu L.N. (2004). Controllable fabrication of fiber nano-tips by dynamic chemical etching based on siphon principle. J. Vac. Sci. Technol. B.

[36] Frens G. (1973). Controlled nucleation for the regulation of the particle size in monodisperse gold suspensions. Nature.

[37] Hu L., Chen Z.Y., Chen N., Zhang W., Zhu H., Liu S.P., Wang T.Y. Preparation of gold colloid and its surface-enhanced Raman scattering properties. Proceedings of the Communications and Photonics Conference and Exhibition.

[38] Savitzky A., Golay M.J.E. (1964). Smoothing and differentiation of data by simplified least squares procedures. Anal. Chem..

